# An Explorative Study of Haemostasis in Canine Steroid-Responsive Meningitis–Arteritis Using Viscoelastic Monitoring

**DOI:** 10.3390/ani16010050

**Published:** 2025-12-24

**Authors:** Kine Bergum Hjellegjerde, Berry Wong, Sophie Wyatt, Elena Scarpante, Patricia Alvarez, Annette Wessmann, Lucy McMahon, Adam Mugford, Josep Brocal

**Affiliations:** 1 Anderson Moores Veterinary Specialists, The Granary, Bunstead Barns, Poles Lane, Hursley, Winchester SO21 2LL, UK; 2 Hawkshead Campus, The Royal Veterinary College, Hawkshead Lane, Hatfield, Hertfordshire AL9 7TA, UK; 3 Dick White Referral Veterinary Specialists, Station Farm, London Road, Six Mile Bottom, Cambridgeshire CB8 0UH, UK; 4 Neurology and Neurosurgery Service, IVC Evidensia, Pride Veterinary Referrals, Riverside Rd, Derby DE24 8HX, UK; 5 Banco de Sangue Animal, Rua de João de Deus, n°741, 4100-462 Porto, Portugal

**Keywords:** SRMA, VCM, dogs, coagulation, haemostasis, haemorrhage

## Abstract

Canine steroid-responsive meningitis–arteritis (SRMA) is a systemic, immune-mediated, inflammatory disease which occasionally leads to spontaneous haemorrhage, both within and outside the central nervous system, as a possible complication. No previous studies have investigated the haemostatic profile in a cohort of dogs with SRMA using viscoelastic monitoring. The aim of this study was to assess haemostatic function in a cohort of dogs affected by SRMA using the Entegrion VCM (Viscoelastic Coagulation Monitor) Vet™ device. This was a multicentre prospective study conducted between April 2023 and April 2025 recruiting dogs with SRMA from four veterinary referral hospitals in the United Kingdom. All four research centres used the Entegrion VCM Vet™ device for evaluation of haemostasis. Twenty dogs were included in the study. One dog had a hypercoagulable VCM result, and two dogs were considered hyperfibrinolytic based on their VCM results. No dogs had any clinical signs of vascular complications (ischaemic and/or haemorrhagic stroke, haematomas, or haemorrhages). Although the pathophysiology of vascular events in dogs with SRMA remains unclear, the results of this study suggest that further investigations into the fibrinolytic system and endothelial structure in dogs affected by SRMA are warranted.

## Simple Summary

Steroid-responsive meningitis–arteritis is an immune-mediated inflammatory disease involving the nervous system in dogs that can occasionally lead to bleeding inside or outside the brain and spinal cord. This study looked for any abnormalities in the blood clotting process using the Entegrion VCM Vet™ device in dogs diagnosed with steroid-responsive meningitis–arteritis. The study was performed at four veterinary hospitals in the United Kingdom between 2023 and 2025. Twenty dogs were included in the study, none of which had obvious bleeding or clotting problems on examination. One dog had blood that had increased likelihood for clotting, and two had blood that broke down clots too quickly when measured using the Entegrion VCM Vet™ device. Although no dogs had any outward signs of bleeding or clotting complications, the findings of this study are important because they suggest that steroid-responsive meningitis–arteritis may affect the blood’s clotting and clot-breaking systems, thereby supporting the need for further investigations into this. Understanding this better could help veterinarians predict, monitor, and prevent potential bleeding or clotting problems in dogs affected by steroid-responsive meningitis–arteritis.

## 1. Introduction

Steroid-responsive meningitis–arteritis (SRMA) is a systemic, non-infectious, immune-mediated, inflammatory disease in dogs, with a reported prevalence of 1.6–2% in referral veterinary hospitals [1,2]. Although the disease can be seen in dogs of any age, it most commonly affects juvenile dogs between the ages of 6 to 18 months [3,4]. Medium- to large-size dog breeds, specifically breeds such as Beagles, Boxers, Bernese Mountain Dogs, Weimaraners, and Nova Scotia Duck Tolling Retrievers, are overrepresented.

SRMA is characterised by neutrophilic leptomeningitis and necrotising arteritis of the associated arteries [5]; however, several studies support a generalised humoral immune response and systemic involvement [5,6,7,8,9,10,11,12]. Any artery in the body may be affected, including arteries of the heart, mediastinum, or thyroid gland [13,14]. Dogs with the acute onset form of SRMA exhibit marked spinal hyperaesthesia, pyrexia, and neutrophilic pleocytosis and increased protein on cerebrospinal fluid (CSF) analysis [5,6,15].

Early and appropriate immunosuppressive treatment is typically associated with a favourable prognosis in dogs with SRMA [1,15]. Immunosuppressive doses of corticosteroids commonly result in marked improvement of clinical signs within days, but treatment duration extends from weeks to months, and relapses can occur [1,4,6,7,9,16].

Spontaneous haemorrhage, both within and outside the central nervous system (CNS), has been reported as a possible complication of SRMA [17,18,19,20,21,22,23], although the underlying pathophysiology remains unknown. Vascular complications secondary to abnormal haemostasis are reported with several systemic inflammatory disease processes such as acute pancreatitis [24], chronic inflammatory enteropathy [25], and immune-mediated haemolytic anaemia [26]. A literature search in the PubMed, ResearchGate, and CABI databases using the search terms “SRMA”, “coagulation”, and “haemostasis” (last searched on 31 October 2025) did not identify any relevant literature that aimed to investigate haemostasis in a cohort of dogs with SRMA using the Entegrion VCM (Viscoelastic Coagulation Monitor) Vet™ (Entegrion, Durham, NC, USA) device.

Viscoelastic testing offers dynamic, real-time analysis of a patient’s coagulation status by quantifying clot initiation, clot kinetics, clot firmness, and fibrinolysis. Historically constrained to specialised laboratories due to equipment costs and technical expertise requirements, recent advancements in point-of-care viscoelastic analysers, capable of utilising untreated whole blood samples, have expanded the accessibility and clinical utility of this technology across veterinary practices [27]. The objective of this study was to assess haemostatic function in a cohort of dogs diagnosed with SRMA using the Entegrion VCM Vet™, a viscoelastic testing device that has been validated for this use in canine patients [28].

## 2. Materials and Methods

This was a multicentre prospective study conducted between April 2023 and April 2025 recruiting patients from four veterinary referral hospitals in the United Kingdom: Anderson Moores Veterinary Specialists (Hampshire, UK); Dick White Referrals (Cambridgeshire, UK); Pride Veterinary Referrals (Derbyshire, UK); and the Royal Veterinary College (Hertfordshire, UK). Dogs were included following diagnosis of SRMA by a board-certified neurologist or internal medicine specialist. A diagnosis was made based on typical clinical signs and examination findings, such as pyrexia (≥39.2 °C), spinal hyperaesthesia, exclusion of other causes that could account for the reported clinical signs, CSF analysis indicating a predominantly neutrophilic pleocytosis with no evidence of degenerate neutrophils or pathologic organisms, and resolution of clinical signs with corticosteroid treatment. Dogs were excluded from the study if they had received antithrombotic, fibrinolytic/antifibrinolytic, or immunosuppressive medications within eight weeks prior to presentation. Patients who did not have a complete blood cell count performed, were thrombocytopenic (platelet count < 66 × 10^9^/L without platelet clumping confirmed by blood film analysis) or had a haematocrit in a range that has been reported to affect haemostasis (<30% or ≥60%) [29] were also excluded from the study. Presence of neurological deficits was not considered an exclusion criterion in order to include dogs with SRMA that developed central nervous system vascular complications [17].

**Figure 1:**
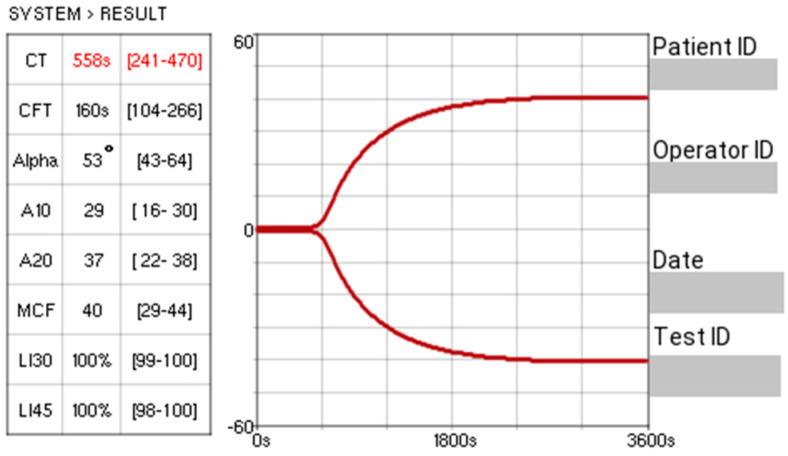
An example trace from Entegrion VCM Vet™ (the value “s” refers to seconds). The red number reflects a result outside the reference range.

All research centres had the same point-of-care viscoelastic monitoring system: Entegrion VCM Vet™, which was used for the evaluation of haemostasis. Quality assurance procedures were conducted in accordance with the manufacturer’s instructions outlined in the device’s operating manual. A sample from each dog was analysed on the Entegrion VCM Vet™ using their standardised operating instructions at the time of presentation. The device analyses stages of coagulation, conducts a value for each set stage, and produces an associated graphical trace, as seen in Figure 1. Six parameters were recorded from the viscoelastic test: clot time (CT); clot formation time (CFT); alpha (α) angle; maximum clot firmness (MCF); Lysis Index 30 (LI30); and Lysis Index 45 (LI45). CT is defined as the interval from the initiation of the test to the point at which the clotting amplitude reached 1% above baseline. CFT is recorded as the time taken for the amplitude to reach 10%, reflecting the progression of clot development. The alpha angle represents the rate of fibrin build-up and cross-linking, providing an indication of clot kinetics. MCF reflects the overall strength and stability of the clot, corresponding to the peak amplitude achieved prior to the onset of fibrinolysis. LI30 and LI45 measure the percentage of clot firmness/strength remaining 30 and 45 min after reaching MCF, respectively, reflecting degrees of fibrinolysis. Accepted reference ranges exist based on previous studies [28,30] and were as follows: CT 241–470 s, CFT 104–266 s, alpha angle 43–64°, MCF 29–44 VCM units, LI30 99–100%, and LI45 98–100%. Device-specific reference intervals were not generated for each individual centre.

Based on a recently published study [30], hypercoagulability was defined as a deviation of 25% or more from established reference intervals [28] in at least two relevant parameters, specifically, decreased CT, increased alpha (α) angle, and increased MCF. Conversely, hypocoagulability was defined as a 25% or greater deviation in at least two parameters in the opposite direction, namely, increased CT, decreased α angle, and decreased MCF. The lysis parameters LI30 and LI45 were considered separately from CT, CFT, α angle, and MCF. LI30 and/or LI45 of <85% was considered an indication of excessive fibrinolysis [31,32].

The following data was gathered for each case: date of presentation; signalment; body weight; presenting clinical signs; the presence/absence of neurological signs on presentation; the presence/absence of any clinical signs of vascular complications (i.e., bleeding, petechiae, ecchymoses, clinical signs of thrombosis); treatment prior to diagnosis; clinicopathological testing results (including complete blood cell count, serum biochemistry, and C-reactive protein (CRP)); CSF results; diagnostic imaging findings; area of acquired CSF; VCM results; treatment following diagnosis; and last recorded outcome. All available categorical and continuous data were collected, anonymised, and compiled in a spreadsheet (Microsoft Excel^®^ 2022; version 2508). Due to the design of the study and sample size, descriptive statistics were applied where appropriate. Continuous variables were reported as a mean or median with corresponding ranges, while categorical variables were presented as percentages.

The study was approved by the RCVS Ethics Review Panel under the reference number 2021-067 and the Royal Veterinary College Clinical Research and Ethical Review board (URN 2024 2244-3). All dogs were treated in accordance with their individual medical requirements, as determined by the attending veterinarians. Written informed consent was obtained from the owners of all dogs for the use of their clinical data in the study.

During the preparation of this manuscript, AI assistance (OpenAI 2023, ChatGPT, GPT-4.1-mini, San Francisco, CA, USA) was limited to improving grammar and readability. All scientific content was solely generated and provided by the authors. The authors have reviewed and edited the output and take full responsibility for the content of this publication.

## 3. Results

### 3.1. Study Population

A total of 20 dogs fulfilled the inclusion criteria and were enrolled in the study between April 2023 and April 2025. There were 9 males (45%; 1 was neutered, and 8 were entire) and 11 females (55%; 3 were neutered, and 8 were entire). Ages ranged from 3 to 37 months (mean of 12.25 and median of 9.5 months). Body weights ranged from 3.1 kg to 58.5 kg (mean of 17.2 kg and median of 14.05 kg). A total of 11 dog breeds were represented, the most common being mixed-breed dogs (8/20, 40%), Miniature Schnauzers (3/20, 15%), and German Shepherds (2/20, 10%). One dog of each of the following breeds was reported (1/20, 5%): Miniature Dachshund; Beagle; Poodle; Border Terrier; Irish Wolfhound; Weimaraner; and Border Collie.

### 3.2. Clinical Findings

Clinical signs at presentation are summarised in Table 1. No dogs presented with neurological deficits, and no dogs were reported to have any signs of vascular complications on presentation.

Eleven dogs (11/20, 55%) were pyrexic at the time of presentation. Rectal temperature ranged from 37.3 °C to 40.4 °C (mean of 39.7 °C and median of 39.4 °C). CRP was measured in all but two dogs and ranged from 27.6 mg/L to 230 mg/L (mean of 114.8 mg/L and median of 98.7 mg/L).

Eight dogs (8/20, 40%) were tested for antibodies for *Anaplasma* spp. and *Borrelia burgdorferi*, *Ehrlichia* spp., and antigens for *Dirofilaria immitis* using the Idexx SNAP 4Dx™ test (IDEXX, Westbrook, ME, USA). All eight dogs tested negative for the mentioned pathogens. Ten dogs (10/20, 50%) were tested for both *Toxoplasma gondii* and *Neospora caninum*. Seven of these (7/10, 70%) were tested by serology, and the remaining three (3/10, 30%) were tested by PCR performed on CSF. One dog (1/20, 5%) was only tested for *Neospora caninum*; this was tested by serology. All dogs tested negative for the mentioned pathogens.

### 3.3. Treatment Prior to Presentation

Eighteen dogs (18/20, 90%) had received medical therapy during the eight weeks prior to presentation. The following medications were reported: meloxicam (12/18); maropitant (9/18); paracetamol (9/18); methadone (7/18); potentiated amoxicillin (5/18); gabapentin (3/18); buprenorphine (2/18); Microlax^®^ enema (2/18); cefuroxime (1/18); carprofen (1/18); cefalexin (1/18); and marbofloxacin (1/18). Two dogs (2/20, 10%) had not received any medical treatment during the eight weeks prior to presentation.

### 3.4. Diagnosis of SRMA

CSF analysis and cytological evaluation were performed and found to be compatible with the diagnosis of SRMA in all cases. Fourteen dogs (14/20, 70%) had cerebellomedullary cisternal CSF collected, three dogs (3/20, 15%) had lumbar CSF collected, and CSF was collected from both areas for three dogs (3/20, 15%). Total nucleated cell count (TNCC) ranged from 7/µL to 8000/µL (mean of 1128.13/µL and median of 150/µL; reference range < 5 cells/µL). CSF total protein (TP) was reported for 16 dogs (16/20, 80%) and ranged from 18.8 mg/dL to 817.3 mg/dL (mean of 133.17 mg/dL and median of 51.15 mg/dL; reference range < 25 mg/dL for cerebellomedullary cisternal CSF and <45 mg/dL for lumbar CSF). Neutrophilic pleocytosis was reported in 15 dogs (15/20, 75%) and 5 dogs were reported to have mixed predominantly neutrophilic pleocytosis (5/20, 25%).

### 3.5. VCM Results

All 20 dogs had VCM performed following the Entegrion VCM Vet™ standardised operating instructions. The VCM results for the six monitoring parameters selected for this study are shown in Table 2. Of the 20 dogs in this study, 1 was classified as hypercoagulable (outlined in blue in Table 2), 17 were classified as normocoagulable, and 2 were classified as hyperfibrinolytic (outlined in green in Table 2).

### 3.6. Treatment Following Diagnosis and Outcome

All dogs survived to discharge. The final follow-up occurred more than four weeks post-diagnosis for 12 dogs. In four dogs, the post-diagnosis follow-up time was 18, 7, 5, and 4 days, respectively. The remaining four dogs were all lost to follow-up on the day following diagnosis. The follow-up times for the three dogs that had haemostatic changes were 5, 44, and 314 days, respectively. No dog was reported to experience neurological deficits or vascular complications during their follow-up period. All dogs were prescribed immunosuppressive doses of prednisolone (mean dose of 2.2 mg/kg once daily), and all dogs were reported to have resolution of clinical signs following initiation of therapy. The most common forms of analgesia prescribed were paracetamol (17/20, 85%), methadone (10/20, 50%), and gabapentin (8/20, 40%). Four dogs (4/20, 20%) were reported to experience relapse of their condition on day 90, 146, 136, and 310 following initial diagnosis, respectively. None of these dogs had abnormal VCM results at initial diagnosis.

## 4. Discussion

This exploratory study is the first to assess the haemostatic function in a cohort of dogs with SRMA using VCM. Three dogs in our cohort were considered as having haemostatic alterations by their VCM results. One dog (Dog 16) was hypercoagulable, and two dogs (Dog 11 and Dog 20) revealed changes supportive of excessive fibrinolysis based on our reference ranges. Dog 16 was a seven-month-old female entire Weimaraner. This patient had received a subcutaneous injection of maropitant ten days prior to presentation but no other therapy. The follow-up period was 314 days, and as with all dogs in this study, there were no reports of vascular events. This patient did not experience any relapses during the follow-up period. This dog’s VCM revealed a CT of 169 s and an MCF of 56, classifying it as hypercoagulable based on our definition and previously published literature [30]. The 25% deviation range for MCF is 21.75–55, just qualifying this patient as having a 25% deviation in this parameter. There is a lack of studies demonstrating what deviation percentages for the VCM parameters, or parameters of other viscoelastic monitoring devices (such as thromboelastography (TEG) and rotational thromboelastometry (ROTEM)), are deemed as clinically significant, making it challenging to interpret mild deviations [33]. There is also no universal consensus, both in veterinary and human medicine, on the definition of hypercoagulability using viscoelastic testing [34]. Hypercoagulability is generally interpreted based on coagulation testing patterns, not strict cutoffs. For the present study, a 25% deviation of two or more VCM parameters was used to define hypocoagulability and hypercoagulability based on a recently published study looking at coagulation status in dogs with immune-mediated polyarthritis (IMPA) [30]. However, the same study also addresses the challenge that comes with the broad variability in the definition of “hypercoagulability” [35]. Two or more coagulation parameters outside of the reference interval have also been used as a definition for hypocoagulability and hypercoagulability in another study examining coagulation status in dogs with naturally occurring *Angiostrongylus vasorum* infection [36].

Both Dog 11 and Dog 20 had LI deviations categorising them as hyperfibrinolytic. Dog 11 was a nine-month-old male entire mixed-breed dog. This patient had received maropitant, buprenorphine, methadone, meloxicam, and potentiated amoxicillin within two days prior to presentation. The VCM trace for this dog revealed a LI30 and LI45 of 54% and 36%, respectively, without any other alterations in the other parameters. The follow-up period was five days, and there were no reports of vascular complications. This patient did not experience any relapses during the follow-up period. Dog 20 was a 19-month-old female entire mixed-breed dog. This patient had received gabapentin, maropitant, methadone, and paracetamol one day prior to presentation. The VCM trace for this dog revealed a LI30 and LI45 of 78% and 56%, respectively, without any other alterations in the other parameters. The follow-up period was 44 days, and there were no reports of vascular events. This patient did not experience any relapses during the follow-up period. As with the case of hypercoagulability, no consensus exists for what is considered a clinically significant deviation from the reference range of the lysis parameters LI30 and LI45 for VCM in animals, and no universal consensus exists on the definition of hyperfibrinolysis using viscoelastic testing [34]. The definition of hyperfibrinolysis used in our study was based on human medicine studies that have shown that a Lysis Onset Time (LOT) of less than 30 min and a LI30 of <85% are indicative of significant fibrinolysis [31,32]. However, it remains unclear whether this can be extrapolated to the canine population. The fibrinolytic system becomes active alongside the coagulation process, serving to maintain haemostatic balance and limit clot formation to sites of vascular damage. It also plays a role in breaking down clots as tissue repair progresses. Imbalance in the fibrinolytic system can lead to either decreased fibrinolysis (hypofibrinolysis) or excessive fibrinolysis (hyperfibrinolysis), which may clinically present as thrombosis or bleeding, respectively [37]. Congenital hyperfibrinolytic disorders are rare in dogs but is reported in Greyhounds [38] and, recently, English Springer Spaniels [39]. Acquired hyperfibrinolysis occurs as a reaction to excessive clot formation and activation of the coagulation system in conditions like disseminated intravascular coagulation (DIC), major trauma, or severe infections, often resulting in consumption of clotting factors and platelets, leading to haemorrhage [37,40]. As an abnormal clot formation is not necessary for abnormal activation of the fibrinolytic system, it is possible to have a normal or near-normal VCM trace on all other parameters except the lysis parameters, as with Dog 11 and Dog 20 in this study. Increased serum and CSF D-dimers, a fibrin breakdown product, has been reported in a case report on a dog with SRMA and concurrent haemorrhagic complications [18]. For this dog, the coagulation markers prothrombin time (PT), activated partial thromboplastin time (aPTT), buccal mucosal bleeding time (BMBT), and TEG were all without abnormalities. CSF D-dimer concentration was also found to be significantly increased in dogs with SRMA in a study looking at fibrinolytic activity in CSF in dogs with different neurological disorders [41]. As D-dimer reflects fibrin breakdown (past or ongoing) but viscoelastic monitoring reflects current clotting dynamics, normal viscoelastic monitoring with increased D-dimers is possible and may reflect previous fibrinolytic activity. None of the dogs in our study had D-dimers performed, which may have been helpful in excluding previous fibrinolysis in the dogs with normal VCM results. On the other hand, hyperfibrinolysis may not always be associated with bleeding. The underlying pathophysiological mechanisms of hyperfibrinolysis in dogs remain poorly understood, and the significance of detecting excessive fibrinolysis in clinically healthy dogs is uncertain [42]. Future studies investigating the fibrinolytic system in dogs with SRMA could be helpful in understanding its possible implications in these patients. All VCM tests in this study were performed on whole blood without the addition of any activators, and future studies may benefit from using tissue plasminogen activator (tPA)-augmented viscoelastic testing to also assess how prone a sample is to breaking down clots [43].

Vascular complications can have detrimental consequences, hence the importance of trying to understand potential underlying aetiologies. A recent paper reported the prevalence of CNS vascular complications in dogs with SRMA to be 21.2% [17], which is in contrast to our study where no dogs were reported to experience such complications. However, the same study only included dogs with advanced imaging, which may have created a bias towards dogs with SRMA that have neurological deficits because a diagnosis of SRMA might not necessarily require advanced imaging. The short follow-up time for eight of the dogs in our study may have influenced our results and decreased the confidence in excluding subclinical vascular complications in these patients. However, other studies [18,19,20,21,22] reported occurrence of vascular complications at the time of presentation, making it likely that we would have registered them even with short follow-up times. As discussed in a previously published study [17], many clinicians still assume that dogs with acute SRMA cannot have neurological deficits and therefore this may have led to a degree of selection bias in our study. Typically, SRMA does not cause any neurological signs, but in the presence of vascular CNS complications, major neurological deficits are likely to be present [17,18,19,20,21,22].

None of the dogs in our study were reported to have vascular complications at the time of presentation or during the follow-up period. Certain subclinical forms of vascular events, such as subclinical thrombosis or subclinical haemorrhage, can be challenging to detect and would usually require advanced imaging to diagnose. None of the three dogs that had abnormal VCM results had advanced imaging performed, and the assumption that they had no vascular complications was made based on their neurological examination and clinical presentation. It is possible that this was not adequate to reach this conclusion. Future studies should explore the options to include advanced imaging of dogs with SRMA, as this may strengthen the interpretation of potential pathophysiological mechanisms. In addition, future studies looking at VCM testing in dogs with SRMA with confirmed vascular complications could provide additional information.

Seventeen dogs (17/20, 85%) in our cohort had normal VCM results. If the same finding was to be present in a larger sample size, global assessment could suggest that dogs with SRMA do not commonly have alterations on VCM. This could potentially suggest that another aetiology for bleeding, such as fragility of blood vessels due to inflammation, may be more likely. A notable limitation of VCM is the inability to assess the contribution of endothelial function in hemostasis [44]. Another limitation of the VCM is its high sensitivity for identifying normocoagulable states (approximately 91–94%), but moderate to poor specificity. Consequently, the VCM may incorrectly classify 23–44% of dogs with abnormal coagulation as normocoagulable [45]. As a result, both hypo- and hypercoagulable states may be missed, and a normocoagulable VCM result should have ideally be confirmed using more established coagulation testing methods (i.e., ROTEM) [45].

The relapse rate in this study was 20%. While this is in accordance with other studies that report a relapse rate between 16 and 60% in dogs with SRMA [4,7,8,9,46,47,48], eight dogs in our study had a very short follow-up time, which may have influenced this finding.

Not all dogs in this study had infectious disease testing performed. However, all dogs were treated with immunosuppressive doses of prednisolone, and all were reported to respond favourably to treatment, making an undetected underlying infectious condition unlikely.

Eighteen dogs had received medical therapy within the eight weeks prior to presentation. Of the medications reported, meloxicam (12/20) and carprofen (1/20) are considered to have a mild potential to alter haemostasis by decreasing platelet aggregation due to COX (cyclooxygenase) inhibition [49]. If, at all, haemostatic alterations are seen secondary to these medications, mild hypocoagulability would be expected, a tendency that was not seen in any of the dogs in this study. Haemostatic complications secondary to meloxicam and carprofen are considered rare and are therefore unlikely to have significantly affected the results of this study [50,51].

One major limitation of our study is the lack of a control group. We based our study on previously published and accepted reference ranges for the VCM device [28]; however, reference ranges are population-wide and not experiment-specific. Reference ranges do not account for study-specific variables, and the lack of a control group therefore does not allow for any statistical comparison in our study. Future studies would benefit from including a control group.

A second major limitation of this study is the lack of concurrent standard coagulation testing, such as PT and aPTT. However, in a recent study looking at nine dogs diagnosed with SRMA and concurrent vascular complications, PT and aPTT were within reference range for all eight dogs tested [23]. In many conditions, changes in PT and aPTT may be reflected in viscoelastic monitoring by clot formation time and clot strength [52,53]. However, there are studies suggesting a substantial discordance between plasma-based clotting times and whole-blood viscoelastic behaviour [54,55,56]. Most of the mentioned literature used TEG devices and not the VCM used in this study, which is a considering factor when assessing the results of our study. Future studies should aim to include standard coagulation testing, as using both approaches may give a more complete picture.

This study also has several other limitations, with one being the small number of dogs included. A larger sample size may have increased the chances of documenting haemostatic alterations or strengthened the evidence for the alterations found in the three dogs in this study. SRMA is reported to have a prevalence of 1.6–2% in referral settings [1,2], but the true prevalence of the disease may be higher. With SRMA being an easily recognisable condition for many clinicians, it is possible that many dogs with SRMA are kept under the care of their primary veterinarian, affecting the number of cases seen at our referral practices. All dogs had only one VCM sample performed, which was run by different operators, with no additional coagulation assays such as fibrinogen or D-dimers performed that may have provided a more complete picture of their respective coagulation profiles. Given that multiple personnel were involved in the collection and handling of blood samples, and even though there was an established institutional protocol for sampling and sample handling, the possibility of analytical errors could not be completely ruled out. No standardised operator training or calibration procedures across the four study centres were performed; however, the manufacturer’s guidelines regarding routine use of quality controls were followed as per Partnership on Rational ViscoElastic Test Standardization (PROVETS) recommendations [34]. In this study, we used the established reference intervals for the Entegrion VCM Vet™ [28], and inter-device agreement was not evaluated. A previous study reported the VCM Vet™ inter-device reference range variability [28]; however, the same study also reported moderate-to-good correlation between VCM Vet™ devices and suggested that the observed variations between the devices were not likely to be of clinical significance [28]. According to the guidelines for establishing reference intervals in veterinary medicine, at least 120 samples should be used to generate a reference interval [57], and this may prove challenging in a clinical setting. Despite this, future studies should aim to evaluate inter-device agreement and establish device-specific reference intervals as per the PROVETS recommendations [34]. This highlights the need for caution when interpreting the values presented in this paper.

## 5. Conclusions

In this explorative study of haemostasis in dogs affected by SRMA, three dogs had VCM results supportive of haemostatic alterations. None of these dogs had any clinical signs of vascular complications. The underlying pathophysiology of vascular events reported in dogs with SRMA remain unclear. However, the results of this study support the need for further investigations into the fibrinolytic system and endothelial structure in dogs with SRMA.

## Figures and Tables

**Table 1:** Clinical signs in dogs with SRMA; *n* = 20.

Clinical Signs	Dogs (%)
Cervical hyperaesthesia	17 (85)
Lethargy	11 (55)
Pyrexia	11 (55)
Thoracolumbar hyperaesthesia	7 (35)
Low head carriage	6 (30)
Stiff gait	5 (25)
Lameness	4 (20)
Hyporexia/Inappetence	4 (20)
Abdominal tension	2 (10)
Panting	1 (5)
Cervical lymphadenomegaly	1 (5)
Discomfort upon lifting the tail	1 (5)
Shivering	1 (5)
Restlessness	1 (5)
Thoracolumbar kyphosis	1 (5)
Weight loss	1 (5)

**Table 2:** The VCM results for the 20 dogs included in the study (the value “s” refers to seconds). Reference ranges: CT 241–470 s; CFT 104–266 s; alpha angle 43–64°; MCF 29–44 VCM units; LI30 99–100%; LI45 98–100%.

Dog	CT (s)	CFT (s)	α Angle (°)	MCF (VCM Units)	LI30 (%)	LI45 (%)
1	421	108	63	44	100	99
2	507	141	57	40	100	100
3	533	163	54	39	100	100
4	588	160	54	40	100	100
5	405	143	56	42	100	99
6	429	105	62	46	100	100
7	372	83	69	57	100	98
8	331	276	33	43	100	100
9	489	130	58	57	100	100
10	445	105	63	51	100	100
11	384	99	63	44	54	36
12	273	149	52	71	100	100
13	407	111	60	57	100	99
14	567	247	39	36	100	100
15	390	132	57	49	100	98
16	169	94	63	56	100	100
17	506	155	48	43	100	98
18	443	155	57	46	100	100
19	302	143	54	50	100	100
20	473	157	55	30	78	56

## Data Availability

Data is contained within the article. The data presented in this study is available in the tables and figures in the article “An Explorative Study of Haemostasis in Canine Steroid-Responsive Meningitis–Arteritis using Viscoelastic Monitoring”.

## Abbreviations

The following abbreviations are used in this manuscript:

SRMA	Steroid-Responsive Meningitis–Arteritis
VCM	Viscoelastic Coagulation Monitoring
TEG	Thrombelastogram
ROTEM	Rotational Thromboelastometry
RCVS	Royal College of Veterinary Surgeons
PCR	Polymerase Chain Reaction
CNS	Central Nervous System
CRP	C-reactive Protein
CSF	Cerebrospinal Fluid
CT	Clot Time
CFT	Clot Formation Time
COX	Cyclooxygenase
MCF	Maximum Clot Firmness
LI	Lysis Index
LI30	Lysis Index 30
LI45	Lysis Index 45
TNCC	Total Nucleated Cell Count
TP	Total Protein
aPTT	Activated Partial Thromboplastin Time
PT	Prothrombin Time
PROVETS	Partnership on Rational ViscoElastic Test Standardization
